# Hearing and Balance in Adult Patients with a History of Traumatic Brain Injury*

**DOI:** 10.1055/s-0046-1817135

**Published:** 2026-03-25

**Authors:** Maria Clara Martins Pace, Ana Karina Lima Buriti, Raquel Caroline Ferreira Lopes Fontanelli, Italo Capraro Suriano, Daniela Gil

**Affiliations:** 1 Department of Speech-Language-Hearing Sciences, Universidade Federal de São Paulo, São Paulo, SP, Brazil; 2 Lauro Wanderley University Hospital, Universidade Federal da Paraíba, João Pessoa, PB, Brazil; 3 Department of Speech, Language and Hearing Sciences, Universidade Federal de São Paulo, São Paulo, SP, Brazil; 4 Neurotrauma Outpatient Center, Hospital São Paulo, Universidade Federal de São Paulo, São Paulo, SP, Brazil; 5 Department of Speech-Language-Hearing Sciences, Universidade Federal de São Paulo, São Paulo, SP, Brazil

**Keywords:** hearing, postural balance, brain injury, screening, adult

## Abstract

**Introduction:**

Traumatic brain injury (TBI) can result in peripheral or central injury, causing hearing loss.

**Objective:**

To identify possible hearing and balance disorders in adult patients with a history of TBI.

**Methods:**

Observational cross-sectional study conducted in a public hospital. The sample consisted of 34 individuals aged 18 to 60 years old who had suffered mild to severe TBI more than 6 months before. They underwent an otoscopy and a medical history survey, recording their clinical history, as well as their previous and current hearing and balance complaints. Next, their hearing was screened at 1,000, 2,000, and 4,000 Hz, and their balance was assessed using the Classic and Sharpened Romberg tests, as well as the Timed Up and Go test.

**Results:**

The present study assessed 34 patients, of whom 35.3% failed the right ear and 38.2% failed the left ear in the hearing screening, highlighting a statistically significant difference between the pre- and post-trauma results. Regarding balance, 3.1% had a positive result on the right and 3.1% positive on the left in the Classic Romberg test. The Sharpened test found a higher result of 9.4 positive on the left and front. The Timed Up and Go test results were normal for 75.0% and abnormal for 25% of the participants, revealing a risk of falling.

**Conclusion:**

The present study demonstrated that adults after TBI may have hearing and balance complaints and changes, highlighting the importance of referral to an otorhinolaryngologist and subsequent clinical diagnosis in this population.

## Introduction

Neurological trauma is one of the public health problems typically associated with contact and inertial force on the brain, being one of the leading causes of morbidity and mortality worldwide, affecting children, adults, and older adults. 1 In the public hospital where the present study was conducted, ∼ 500,000 people require hospital care annually due to traumatic brain injury (TBI). Of these, 15 to 20% die within a few hours after the trauma, while another 15% develop irreversible loss of some neurological function. 2 Traumatic brain injury is responsible for high mortality rates and is more prevalent in young males. 3 4 5

Traumatic brain injury has been identified as one of the main causes of mortality and morbidity, with a major economic impact worldwide. A study 6 demonstrated that even brief episodes of hypoperfusion and hypoxia can cause injury secondary to TBI and lead to worse short-term and long-term functional loss outcomes. Therefore, the authors 6 highlighted that TBI treatment should involve the prevention of secondary insults.

Car accidents (50%) are among the main causes of TBI. The main age groups among these patients are adolescents and young adults (15–24 years old), and falls are responsible for 30% of traumas. Moreover, many older people are among those who suffer falls. However, in the public hospital where the present study was conducted, falls from slabs are frequent, which are ignored by international statistics. Violence-related causes, such as injuries from firearm projectiles and sharp weapons, account for 20% of traumas. Therefore, TBIs are a relevant public health issue in today's society, with consequences that go beyond medical limits, given their socioeconomic implications. 7

Dizziness and imbalance can be post-TBI sequelae, especially when the trauma results in cerebellar injury. Vestibular system disorders involve continuous and intermittent dizziness and reduced body balance, increasing the risk of falls. These changes in body balance can be secondary to impairments in the interaction between the sensory and motor systems involving the vestibule (responsible for the position and movement of the head) and proprioception (related to posture and body movement). These systems allow the body to remain still, stable, or to move harmoniously. In the absence of a functional vestibular system, the central nervous system (CNS) has difficulty integrating information adequately from the visual and proprioceptive systems. 8

Hearing screening is a quick and effective way to address suspected hearing loss in people who have difficulty with their activities of daily living, aiming at rehabilitation for hearing impairment. 9

The different possible neurological complications (type and location of injury, presence or absence of subdural hematoma, increased intracranial pressure) and the combined personal and environmental factors make each case of TBI unique in its manifestations. Hence, a complete assessment is necessary, verifying all the probable difficulties of the patient, to create a good rehabilitation program.

Thus, the present study aimed at identifying possible hearing and balance changes in adult patients with a history of TBI.

## Methods

The present cross-sectional observational study was carried out at the neurotrauma/neurosurgery outpatient clinic of Hospital São Paulo and Department of Speech, Language and Hearing Sciences. It began after approval by the Research Ethics Committee of UNIFESP – Universidade Federal de São Paulo under number 0435/2019. All participants signed an informed consent form.

All study subjects were being followed at the neurosurgery and neurotrauma outpatient clinic of Hospital São Paulo. The study selected 42 individuals, but 8 were excluded for having different etiologies, being diagnosed with TBI < 6 months before, or not having undergone cranioplasty. Thus, the sample had 34 individuals with a history of mild to moderate TBI according to the Glasgow scale (score of 9–15), of both sexes, aged 18 to 60 years old. The study procedures were performed during the patients' interconsultation at the neurology outpatient clinic.

**Fig. 1:**
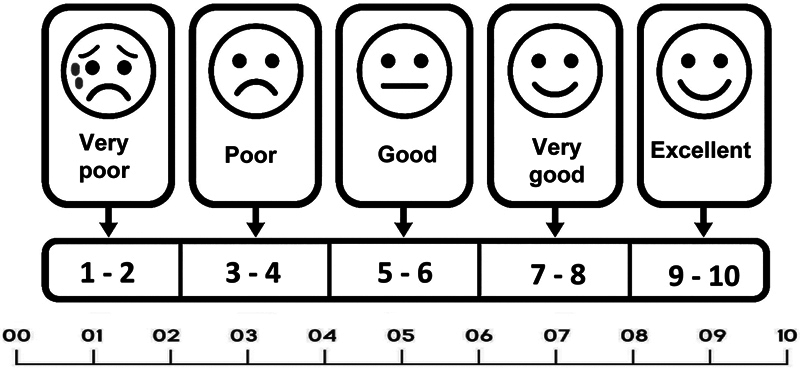
Visual analog scale.

The participants underwent medical and clinical history surveys and then assigned scores from 1 to 10 on a visual analog scale (VAS) ( Fig. 1 ) to classify the self-perception of hearing before and after the injury, with (1) being equivalent to very poor and (10) to excellent. Next, otoscopy and hearing assessment were performed with the hearing screening proposed by the Guidelines for Audiologic Screening, 9 a Maico MA 41 clinical audiometer (Maico), and calibrated TDH earphones (Telephonics). The test was performed in a quiet room, with the individual facing away from the evaluator. After positioning the earphones, they were instructed to raise the hand corresponding to the tested ear when they heard the whistle, even if at a low intensity. Prior training was performed at a comfortable hearing level to check whether they understood the procedure. Then, pure tones were presented at 25 dBHL at 1,000, 2,000, and 4,000 Hz, first in the ear in which the individual reported having better hearing and then in the opposite ear.

After the hearing screening, their static balance was assessed with the Classic and Sharpened Romberg Tests, 10 and their dynamic balance, using the Timed Up and Go (TUG) test. 11

The Romberg tests assessed static balance and verified proprioception – that is, the body's ability to perceive the position and movement of its parts. The Classic Romberg test was performed with the patient standing barefoot, feet together, and arms at the side of the body. They were asked to remain upright for 1 minute, feet together, and looking forward. Then, the same action was requested, but with eyes closed, for 20 to 30 seconds. The Sharpened Romberg test was performed with a cushion on the floor, asking the participant to stand on the cushion with both feet together and remain upright for 1 minute, initially with eyes open and then with eyes closed.

The TUG assesses dynamic balance, measuring mobility and evaluating the risk of falling. It suggests the need for interventions to improve physical performance and reduce the risk of fractures. The examiner explained to the individual that they should walk to the line marked on the floor and return at their own pace. This test timed how long they took to get up from a chair (without armrests), walk 3 meters, turn around, walk back to the chair, and sit down again. At the end, the time spent performing the activity was recorded. The shorter the time used, the better the performance in the test.

To analyze the results, the hearing screening was analyzed according to the pass-fail criterion, 9 in which the individual passes when responding at 75% of the frequencies at 25 dBHL, and fails when not responding in ≥ 2 frequencies. The results were recorded with the letters “P” for “pass” and “F” for “fail”.

For the balance assessment results, vestibular asymmetry was considered positive if the patient displaced their feet or fell in the Classic Romberg test. The Sharpened Romberg Test with a cushion was considered positive when the posture was stable with the eyes open, but not with the eyes closed. The Classic and Sharpened Romberg tests were recorded as “normal” or “positive/negative, and fall to the right or left side” 10 .

For the TUG, those who completed the test in > 12.3 seconds were classified as having an increased risk of falls. The results and displacement time were recorded in the protocol.

Descriptive statistics were used for numerical data analysis, with summary measures (position and variability) such as mean and standard deviation, and relevant statistical graphs to visualize the results adequately. Numerical data were compared with inferential statistics, using the Student's t-test for dependent and independent samples. Categorical data were compared with nonparametric statistics using the Pearson's Chi-squared association test or the extended Fisher's exact test. Significance was set at 5% ( *p* < 0.05) for the analysis of statistical significance, represented in bold and marked (*) in the tables.

## Results

The present study included 34 individuals with mild to moderate TBI, with a median age of 37 years old (standard deviation [SD]: 13.3), as described in Table 1 regarding sex and the causes and location of the TBI. Particularly concerning location of TBI, there was a wide range of brain regions affected, with the highest incidence in the right and left frontal, right temporal, and right parietotemporal regions.

**Table 1:** Sample characterization ( *n* = 34)

Variable		Frequency ( *n* )	Percentage (%)
**Sex**	Female	4	11.8
	Male	30	88.2
**Age (years old)**	18–29	10	29.4
	30–60	24	70.6
**Cause of the lesion**	Car accident	14	41.2
	Hitting the head against a stationary object	8	23.5
	Fall	12	35.3
**Place of the lesion**	Frontal R and L	6	17.6
	Frontal R	3	8.8
	Frontal L	2	5.9
	Fronto-parietal L	1	2.9
	Fronto-parietal R	1	2.9
	Parietal R	1	2.9
	Parietal R and L	2	5.9
	Parietal L	1	2.9
	Occipitoparietal L	1	2.9
	Parietooccipital R	1	2.9
	Parietotemporal R	4	11.8
	Parietotemporal L	2	5.9
	Temporal R	5	14.7
	Temporal L	1	2.9
**Total**		**34**	**100**

**Abbreviations:** L, left; R, right.

Table 2 shows the hearing screening results of the right and left ears according to the pass-fail criterion, distributed by frequency and corresponding percentage.

**Table 2:** Description of hearing screening results

HEARING SCREENING		Right ear		Left ear	
Results		*n*	%	*n*	%
Valid	Obstructed EAC	1	2.9	0	0
	Fail	12	35.3	13	38.2
	Pass	21	61.8	21	61.8
	**Total**	34	100.0	34	100.0

**Abbreviation** : EAC, external auditory canal.

Altogether, 35.3% ( *n* = 12) individuals in the sample failed at least 1 ear, and 61.8% ( *n* = 21) passed the hearing screening bilaterally ( Table 2 ). Also, 2.9% ( *n* = 1) had an obstructed external auditory canal due to the presence of wax, specifically in the right ear.

Table 3 describes the results of the individuals' self-assessment through the pre- and post-TBI scores, which they attributed based on the VAS ( Fig. 1 ). The pre-TBI result had a mean of 9.02, and the post-TBI results had a mean of 7.7, with a statistically significant difference of *p* < 0.001 between the means.

**Table 3:** Descriptive measures of auditory self-perception ( *n* = 34)

Auditory self-perception								
Results	Mean	Median	Standard deviation	Minimum	Maximum	Confidence interval (lower)	Confidence interval (upper)	*p-value* *
Pre-TBI scores	9.029	10.000	1.5856	4.0	10.0	8.441	9.500	** < 0.001**
Post-TBI scores	7.706	8.000	2.1253	2.0	10.0	6.971	8.353	

**Abbreviation** : TBI, traumatic brain injury.

**Note** : *Student's t-test: 5% significance level ( *p* < 0.05).

Table 4 presents the descriptive measures of the comparison between the scores they gave and the hearing screening results for the right and left ears.

**Table 4:** Descriptive measures of the comparison between the scores given and the hearing screening results for the right and left ear

Hearing Screening	Right ear				Left ear			
Results		*n*	Mean	Standard deviation		*n*	Mean	Standard deviation
Pre-TBI scores a	Pass	21	9.238	1.2209	Pass	21	8.810	1.6917
	Fail	12	8.583	2.1088	Fail	13	9.385	1.3868
Post-TBI scores b	Pass	21	8.333	1.5599	Pass	21	7.714	2.0284
	Fail	12	6.667	2.6742	Fail	13	7.692	2.3588

**Abbreviation** : TBI, traumatic brain injury.

**Notes: Student's t-test for independent samples:**

a Pre-TBI scores and screening results RE: *p* = 0.265; Pre-TBI scores and screening results LE: p = 0.311;

b Post-TBI scores and screening results RE: *p* = 0.066**; Post-TBI scores and screening results LE: p = 0.977.

There was a difference (although not statistically significant) between the mean pre-TBI and post-TBI scores of those who passed the hearing screening in relation to those who failed in the right ear ( Table 4 ). There was no statistically significant difference between the mean pre-TBI and post-TBI scores of those who passed the hearing screening in relation to those who failed in the left ear.

Only 32 individuals were included in the static and dynamic balance assessment ( Table 5 ), as two participants were in wheelchairs, preventing them from performing the locomotion movements required in the tests.

**Table 5:** Description of the results of the static and dynamic balance tests ( *n* = 32)

Balance tests	Results	Frequency ( *n* )	Percentage (%)
Classic Romberg			
	Negative	30	93.8
	Positive R	1	3.1
	Positive L	1	3.1
**Sharpened Romberg**			
	Negative	23	71.9
	Positive R	2	6.2
	Positive L	3	9.4
	Positive forward	3	9.4
	Positive backward	1	3.1
**Timed Up and Go (TUG)**			
	Abnormal	8	25.0
	Normal	24	75.0
**TOTAL**		**32**	**100**

**Abbreviations** : L, left; R, right.

Table 5 presents the static and dynamic balance test results, distributed by frequency and percentage.

The Classic Romberg test had 30 (93.8%) individuals with a negative result, one (3.1%) with a positive result with a deviation to the right, and one (3.1%) with a positive result with a deviation to the left. The Sharpened Romberg test had 23 (71.9%) individuals with a negative result and 9 (28.1%) with a positive result.

As for the dynamic balance test, the TUG results showed that 24 (75%) individuals were within the expected normal range, and 8 (25%) had changes, revealing a risk of falling. The average time of those who performed the test was 12.05 seconds, with a minimum time of 8 seconds and a maximum of 30 seconds ( Table 5 ).

## Discussion

The sample of the present study is consistent with several previous studies, 3 4 5 7 12 whose sample had predominantly young male adults who suffered TBI in a car accident.

Table 2 shows that less than half of the individuals failed bilaterally, demonstrating that hearing screening can be an important primary prevention action. This percentage highlights the need for secondary prevention action, referring individuals who failed for a diagnostic evaluation. Hearing problems can be prevented or improved if a hearing disorder, deficiency, or disability is detected and treated early. 9

In their study, Koohi et al. 13 screened hearing with portable equipment and applied two self-assessment questionnaires (the Amsterdam Inventory Auditory for Disability [AIAD] and the Hearing Handicap Inventory for the Elderly [HHIE]) to poststroke patients. They found hearing loss in 100% of the sample when the hearing screening result was combined with one of the questionnaires.

Costa-Guarisco et al. 14 corroborate the findings of the present study, screening older adults' hearing and assessing their hearing self-perception with a subjective scale of faces. The ability to represent their hearing difficulties equaled the hearing screening results, evidencing agreement between objective and subjective procedures.

The present study used a visual analog scale ranging from 1 to 10, assigning scores for pre-TBI and post-TBI hearing. It identified a pre-TBI mean of 9.02 and a post-TBI mean of 7.7. This result was statistically significant ( *p* < 0.001), demonstrating a difference for the worse in the person's subjective perception of hearing. This indicates the importance of including audiological evaluation in the list of care for post-TBI patients.

The present study compared the self-perception of individuals who suffered TBI with the behavioral assessment that depended on their response to pure tones by frequency. It found that individuals who passed the hearing screening in the right ear obtained higher scores than those who failed, and the differences were significant. Those who failed bilaterally had a higher mean score before TBI than those who passed, but without a statistically significant difference. Therefore, the findings of the present study demonstrate that patients generally felt that their hearing was worse after suffering TBI. This report should be valued for physicians to refer such patients for specific evaluations.

The World Health Organization (WHO) 15 warned that nearly 2.5 billion people worldwide, or 1 in 4 people, will have some degree of hearing loss by 2050. It also highlighted that clinical screening is the strategic point to ensure early identification of hearing loss and hearing diseases at any age. Therefore, it is important to include hearing screening in highly vulnerable outpatient clinics or in remote areas, such as the neurotrauma outpatient clinic where the present study collected data. Notably, 35.3% of individuals failed the post-TBI hearing screening, and the rest generally reported feeling that their hearing was worse after the TBI. Hence, the participants of the study were referred early to hearing diagnostic centers.

A study by Bansal et al. 16 revealed some types of hearing loss after mild head trauma, emphasizing that all patients should have their hearing assessed to detect subclinical hearing loss. Chaitanya et al. 17 observed 56 (93.3%) individuals with hearing loss, mostly with conductive (83.3%) and mixed hearing loss (10%). They also highlighted a possible improvement in the degree of hearing loss over time.

The lack of validated treatments for mild TBIs/concussions does not eliminate the need for follow-up. 18 There is increasing evidence that mild TBIs/concussions can generate long-term consequences, such as functional deficits that limit return to work and a variety of neurocognitive and neuropsychiatric symptoms.

Buriti et al. 19 identified cognitive changes in individuals with mild TBI and proposed intervention through acoustically controlled auditory training. In another study, Buriti et al. 20 also observed that the training program helped individuals with mild TBI to reduce the difficulty in processing acoustic information in the long term, with improved cognitive, memory, and attention skills.

Most individuals had adequate results in the Classic Romberg static balance assessment test, but the Sharpened Romberg test importantly increased the number of patients with abnormal results. When the result suggests changes (that is, when it is positive), it implies that the individual depends on vision to maintain body balance. 21

The present study found changes in some individuals with TBI using the Classic Romberg test, in which only one individual showed changes to both the right and left. In the Sharpened Romberg test, three individuals had changes to the left and two to the right, totaling five failures. In a study by Jalali et al., 22 the Romberg test helped to identify adults with balance problems, finding a high incidence of falls and postfall complications in adults and older adults.

The TUG assessed dynamic balance, finding a mean time of 12.05 seconds, with a minimum of 8 seconds and a maximum of 30 seconds. Thus, 23.5% of the individuals were at risk of falling ( Table 5 ). Furthermore, an increased risk of falling may indicate a risk of a new TBI.

According to a study by Jeong et al., 23 the TUG may be an indicator of risk for falls, as it assesses the risk of fractures and suggests the need for interventions to improve physical performance and reduce the risk of fracture, especially in older adults. Yingyongyudha et al. 24 found that the TUG did not obtain significant results in distinguishing patients with and without a history of falls, correlating with a study by Schoene et al. 25 that indicated moderate predictive capacity for the diagnosis, distinguishing older people with a history of falls.

Studies evaluating balance in post-TBI patients are scarce in the literature. However, Sousa et al. 26 reported that dizziness and imbalance may be sequelae that accompany these patients. A case study of a patient who suffered TBI highlighted complaints such as imbalance, sporadic dizziness, and subjective vertigo in situations of rapid head movements and when standing up quickly, among other symptoms such as headache, nausea, sensation of ear fullness, and tinnitus. On the other hand, the patient recovered their basic daily activities after a program that rehabilitated the labyrinthine system.

In the older population, the younger the age, the better the performance in the TUG balance tests. 27 Therefore, balance worsens, and physical and functional limitations appear with increasing age, suggesting that balance training should be prioritized.

It is worth noting that all individuals in the present study who failed the hearing screening and/or had abnormal body balance results were referred for specific diagnostic evaluations. Moreover, the present research opted for procedures based on the possibility of the neurologist using them in consultation, considering physical space and time for execution.

Referring post-TBI patients for audiological and body balance assessment can significantly help identify deficits in this regard and, above all, improve their quality of life.

Despite the referrals, the lack of comparison between screening results and diagnostic assessments can be considered a limitation of the study. A second limitation would be the failure to exclude individuals with a previous history of dizziness to control the complaint. Therefore, it was not possible to conclude whether the loss of balance was due to TBI or pre-existing conditions. Further studies controlling the variables indicated here are needed to increase evidence on this important topic.

## Conclusion

The results of the hearing screening and body balance assessment in adults who suffered a TBI indicate changes in hearing and balance, highlighting the importance of referral to an otorhinolaryngologist and subsequent clinical diagnosis to direct adequate therapeutic planning for those who failed the screening, thus helping them to improve their quality of life.
